# Exercise-Induced Tendon and Bone Injury in Recreational Runners: A Test-Retest Reliability Study

**DOI:** 10.2196/resprot.4585

**Published:** 2015-10-07

**Authors:** Renae Domaschenz, Nicole Vlahovich, Justin Keogh, Stacey Compton, David C Hughes, Collaborative Research Network For Advancing Exercise & Sports Science Scientific Committee

**Affiliations:** ^1^ Department of Sports Medicine Australian Institute of Sport Australian Sports Commission Bruce Australia; ^2^ John Curtin School of Medical Research Australian National University Acton Australia; ^3^ Faculty of Health Sciences and Medicine Bond University Robina Australia

**Keywords:** exercise, genetics, injury, reliability, survey

## Abstract

**Background:**

Long-distance runners are prone to injuries including Achilles tendinopathy and medial tibial stress syndrome. We have developed an Internet comprehensive self-report questionnaire examining the medical history, injury history, and running habits of adult recreational runners.

**Objective:**

The objective of the study was to evaluate two alternative forms of test-retest reliability of a comprehensive self-report Internet questionnaire retrospectively examining the medical history, injury history, and running habits among a sample of adult recreational runners. This will contribute to the broad aims of a wider study investigating genetics and running injury.

**Methods:**

Invitations to complete an Internet questionnaire were sent by email to a convenience pilot population (test group 1). Inclusion criteria required participants to be a recreational runner age 18 or over, who ran over 15 km per week on a consistent basis. The survey questions addressed regular running habits and any injuries (including signs, symptoms, and diagnosis) of the lower limbs that resulted in discontinuation of running for a period of 2 consecutive weeks or more, within the last 2 years. Questions also addressed general health, age, sex, height, weight, and ethnic background. Participants were then asked to repeat the survey using the Internet platform again after 10-14 days. Following analysis of test group 1, we soft-launched the survey to a larger population (test group 2), through a local running club of 900 members via email platform. The same inclusion criteria applied, however, participants were asked to complete a repeat of the survey by telephone interview after 7-10 days. Selected key questions, important to clarify inclusion or exclusion from the wider genetics study, were selected to evaluate test-retest reliability. Reliability was quantified using the kappa coefficient for categorical data.

**Results:**

In response to the invitation, 28 participants accessed the survey from test group 1, 23 completed the Internet survey on the first occasion, and 20 completed the Internet retest within 10-21 days. Test-retest reliability scored moderate to almost perfect (kappa=.41 to .99) for 19/19 of the key questions analyzed. Following the invitation, 122 participants accessed the survey from test group 2, 101 completed the Internet survey on the first occasion, and 50 were randomly selected and contacted by email inviting them to repeat the survey by telephone interview. There were 33 participants that consented to the telephone interview and 30 completed the questionnaire within 7-10 days. Test-retest reliability scored moderate to almost perfect for 18/19 (kappa=.41 to .99) and slight for 1/19 of the key questions analyzed.

**Conclusions**

We successfully developed a self-reported, retrospective questionnaire, delivered using Internet software, providing stable and reliable answers. We demonstrate that our survey provides a relatively quick, easy to complete, and cost effective method to collect epidemiological data from recreational runners and evaluate these participants for inclusion into a genetic study.

## Introduction

### Identification of Risk Factors for Long-Distance Runners

Several systematic reviews have examined the incidence rates and risk factors of running related injuries [1-4]. Such injuries are common, following a systematic review of the literature, Tonoli et al [4] state that injury incidence varies between 0.1% and 2.6% (*P*<.05). The most common injuries sustained by long-distance runners were found to be Achilles tendinopathy, iIliotibial friction syndrome, and medial tibial stress syndrome [4]. The identification of risk factors for these injury types in runners remains controversial, with conflicting results published in the literature [1,2,5-7].

There are two types of injury that are commonly reported in association with running and exercise: bone stress injuries (including medial tibial stress syndrome and tibial stress fracture) and tendon injuries (including tendinopathy and tendon rupture) [1,6]. In the case of tendon injuries, genetic risk factors have been identified that correlate with increased risk of, or protection from, injury [8]. It has been acknowledged that further investigation is required to confirm and correctly interpret the association of identified polymorphisms with specific injuries [8].

### Developing the Internet Study

Published genetic studies on exercise-induced injuries primarily focus on soft tissue injuries [9], and there is only one study demonstrating association between genetic polymorphisms and increased risk of bone stress injuries [10]. In an attempt to address this, we have designed a study to analyze Australian runners using genome-wide association techniques to identify gene variants that contribute to increased risk of, or protection from, Achilles tendinopathy and/or bone stress injuries. To achieve a large sample size, the study must provide a reliable and convenient method of data collection from prospective participants, allowing them to participate in the research from home and with their own device. We therefore developed an Internet activity survey to prospectively recruit participants relevant to our test groups. Retrospective surveys of sporting injuries have been shown to have some limitations for injury epidemiology research because of issues with injury recall [11,12]. In order to counteract these issues, participants were only asked to recall injuries that had occurred in the previous 2 years.

Surveys should be evaluated for reliability, a critical measurement property for health-related and physical activity variables. Reliability refers to the consistency of answers obtained by the same participant when a measurement is repeated on different occasions [13]. Test-retest reliability is measured by having the same participants complete a survey at two different points in time to see how stable the responses are. Consistency is then quantified with a kappa coefficient statistic for categorical data [14].

Internet survey instruments are increasingly being used as a preferred method of conveniently collecting data from a wide range of participants from various locations. The reliability of survey instruments delivered on the Internet has been previously demonstrated, showing that in the instance of retrospective tobacco exposure and risk, this method of data collection is reliable [15]. Furness et al [16] also demonstrated success using an Internet survey instrument for the study of injuries collected in a retrospective manner in surfing athletes, whereby they recruited 1348 surfers. This high level of response enabled the researchers to produce a comprehensive dataset that is the largest study of surfing injuries in Australia [16].

The purpose of the study reported here is to test the test-retest reliability of an Internet survey platform from a convenience population of first responders, and pilot test participants who accepted an invitation to describe their recreational running epidemiology. This will contribute to the broad aims of the wider study, which are to recruit thousands or even tens of thousands of individuals to investigate injury epidemiology across a wide group of recreational runners in order to gain insight into how a variety of demographic factors may influence injury epidemiology; and to perform genetic assessments for the injury risk factors in a small subsample of the overall injury epidemiology study.

## Methods

### Self-Report Questionnaire

The questionnaire administered was designed to enable recruitment as part of the wider study entitled, “The Genetics of Exercise-Induced Injuries in Tendon and Bone.” This study has very broad aims in the range of wanting to recruit very large numbers of participants to provide injury epidemiology among recreational runners, as well as performing genetic assessments for the injury risk factors in a smaller subsample of the overall injury epidemiology study.

The platform used to deliver the survey was the Internet and commercial survey software (SurveyGizmo). The Internet survey was advertised through appropriate forums relevant to the intended population to analyze sporting injuries and training practices. Participants provided us with data on their demographics and running injuries, but the survey provides a platform for determining whether a participant met the wider genetics study’s inclusion and exclusion criteria. Participation was entirely voluntary and required consent to participate in the research processes described by checking a required “I ACCEPT” box necessary to access the questionnaire. Further information about the project was made available via a check box diverting the participant to another screen with all relevant information and contact details for further clarification if required.

On accepting conditions of participation in this project, participants were taken to the questionnaire and instructed that it would take no more than 30 minutes to complete. The software used allowed participants to exit the survey at any time and complete at a later date, allowing participants to provide their data at the time most suitable to them. A questionnaire was deemed complete when the participant had answered all required questions (customized within the software) and participants had submitted their questionnaire by checking the “SUBMIT” tab inserted at the end of the questionnaire.

The questionnaire developed here combined new items of assessment with adapted versions of the SCOFF questionnaire for examining eating disorders [17]. The full survey is provided in Multimedia Appendix 1. Regular running habits were assessed across several categories including how many years participants had been running for, how many kilometers (km) per week they ran, what terrain running was performed on, and whether orthotics were worn during running. In addition, participants reported any other sports or intentional exercise they participated in regularly over the last 2 years and were specifically asked to report any injuries of the lower limbs within the last 2 years that resulted in discontinuation of running for a consecutive period of 2 weeks or more. This was further examined by asking the participants to report on the signs and symptoms of the injury, how the injury was diagnosed, and whether it was an Achilles tendon or bone stress injury. They were also asked to stipulate any previous hip, knee, or ankle surgery over their lifetime.

In addition, participants were asked to report on their general health, provide any knowledge of existing conditions or diseases, and any known antibiotic or prescribed drug administration. They were asked to describe their regular dietary practices including sport supplement use, as well as any body weight fluctuations. Participants were also asked to report their age, sex, height, weight, and ethnic background to collect demographic data for further epidemiology analysis.

Some of the key variables, important to clarify inclusion or exclusion from the wider genetics study previously mentioned, were then selected for test-retest reliability here (Table 1). Questions of interest included those that addressed diagnosis of the injuries, as these variables may result in less than optimal reliability due to the retrospective design of the study [11]. Several questions from the tail end of the survey were also selected to assess if respondent fatigue had an effect on reliability. To approach this experimentally, we used a test-retest reliability design. This study was approved by the Human Research Ethics Committee of Bond University and the Australian Institute of Sport (RO1688B).

**Table 1:** Questionnaire items tested for reliability.

Questionnaire item		Possible responses
**Running habits and running injuries**		
	How many years have you been running on a regular basis? Regular is defined as at least weekly.	<1/1/2/3/4/5/6/7/8/9/10+
	On average, how many kilometer per week would you run?	<15/15-20/20-30/30-40/40-50/50-60/60+
	What type of terrain is the majority of your running performed on?	Bitumen/cement/hard dirt or gravel/soft dirt/grass/synthetic/treadmill
	In the last 2 years, have you participated in any other sports or intentional exercise on a regular basis (eg, weekly during at least one season)?	Yes/no
	In the last 2 years, have you had any injuries of the lower limbs, which have forced you to discontinue running for a period of 2 weeks or more?	Yes/no
	If yes, how many lower limb injuries have you been diagnosed with in the last 2 years?	1/2/3/4+
	Was the injury diagnosed by a professional doctor?	Yes/no
	Was the injury diagnosed by a professional physiotherapist?	Yes/no
	Was the injury an Achilles tendon injury?	Yes/no
	Was the injury a bone stress injury below the knee?	Yes/no
	Have you ever had hip, knee, or ankle surgery?	Yes/no
	While running do you wear orthotics?	Yes/no
**General health**		
	Have you ever smoked cigarettes?	Yes/no
	To your knowledge, have you ever been treated using quinolone antibiotics (eg, ciprofloxacin, norfloxacin)?	Yes/no/unsure
	To your knowledge, have you ever been treated using corticosteroid medication (eg, cortisone injection, prednisone tablets, prednisolone tablets, flixotide inhaler, pulmicort inhaler, QVAR inhaler, seretide accuhaler, symbicort turbuhaler, steroid cream)?	Yes/no/unsure
	To your knowledge, have you ever been treated using calcium tablets as prescribed by a medical doctor or taken it without a prescription?	Yes/no/unsure
	To your knowledge, have you ever been treated using vitamin D supplementation as prescribed by a medical doctor or taken it without prescription?	Yes/no/unsure
	Do you follow a gluten-free diet?	Yes/no
	Do you have any food allergies or avoidances?	Yes/no

### Participants and Procedures

#### Test Group 1

Initial recruitment involved inviting a convenience sample of participants into a pilot group. Invitation to complete “The Genetics of Exercise-Induced Injuries in Tendon and Bone Study” survey was via email platform including an outline of the purpose of the study and providing potential participants with a link to the Internet survey. Participants were recruited from within our association or among associated colleagues. Inclusion criteria for participants included being a recreational runner aged 18 or over, who ran over 15 km/week on a consistent basis.

In response to the invitation, 28 participants accessed and consented to the conditions of the study as logged by SurveyGizmo. Of these participants, 23 completed the first survey on the first occasion, and after 10-14 days were invited by email to repeat the survey for a second time, 20 completed within 10-21 days after the date of the first completion. This group was deemed test group 1 and were subjected to the Internet-Internet test-retest method.

#### Test Group 2

After data from test group 1 suggested that reliability was more than moderate for 19/19 of the variables analyzed, we then sought to maximize the variability in response and strength in reliability of our questionnaire by soft launching the survey to a wider population using a different test-retest method. We invited a larger convenience sample of participants by promoting the wider genetics study through a local running club of 900 members via email platform as described earlier for group 1. The same inclusion criteria were applied and participant information was provided at the commencement of the survey, and checking the box “I ACCEPT” resulted in access to the Internet survey and provided informed consent.

In response to the invitation, 122 participants accessed and consented to the conditions of the study as logged by SurveyGizmo. Of these participants, 101 completed the first survey on the Internet on the first occasion. Of these participants, 50 were randomly selected and contacted by email platform inviting them to repeat the survey by telephone interview. In response to this invitation, 33 participants agreed to the repeat by telephone interview within 7-10 days of Internet completion and 30 answered the call within 14 days and completed the questionnaire on the second occasion. This group was deemed test group 2 and participants were subjected to the Internet-phone test-retest method.

### Data Analyses

Descriptive statistics were used to describe the cohort characteristics of participants for each group, and reported as a percentage (%) of total participant number within the group illustrated. Some of the key variables important to clarify inclusion or exclusion from the wider genetics study previously mentioned were then selected for test-retest reliability and scored using the kappa statistic, with asymptotic standard error [14]. Reliability was then rated using the scale by Landis and Koch for the purposes of comparing the reliability of key questions [14]. Reliability was rated as poor (below .00), slight (.00-.20), fair (.21-.40), moderate (.41-.60), substantial (.61-.80), or almost perfect (.81-1.00). Data were collected using SurveyGizmo and analyses were conducted using SPSS Statistics version 22.0.

## Results

### Self-Report Questionnaire Scores

This self-report questionnaire worked toward the development of a standardized instrument via which participants not only provide us with data on their demographics and running injuries, but also provide us with a platform for determining whether interested participants meet the wider genetics study’s inclusion criteria. This study analyzed the test-retest reliability of key inclusion/exclusion criteria for the wider genetics study, collected with the developed survey involving the 2 groups. The choice of different methods for test-retest analysis of each group is discussed in the “Methods” section. The results of both approaches scored using the kappa statistic, with asymptotic standard error, the valid number of responses, and number of response options, are highlighted here.

### Cohort Characteristics

There were 20 participants that fulfilled group 1 criteria by completing the survey on the Internet then again on the Internet within 21 days after the first completion. Participants were between 18-68 years of age, with an equal distribution of males (50%, n=10) and females (50%, n=10). As much as 45% (9/20) of participants recorded that they had been running for 6 years or less and the remaining 55% (11/20), 8 years or more. Their running habits were variable, with 50% (10/20) predominantly running 15-30 km/week, 25% (5/20) running 30-40 km/week, and 25% (5/20) running 40 km or more per week (Table 2).

**Table 2:** Cohort characteristics.

Characteristics		Total participants		Test group 1 Internet-Internet		Test group 2 Internet-phone	

		N=50		N=20		N=30	
		n	%	n	%	n	%
**Sex**							
	Male	26	52	10	50	16	53
	Female	24	48	10	50	14	47
**Age (years)**							
	18-25	8	16	4	20	4	13
	26-35	19	38	8	40	11	37
	36-40	6	12	4	20	2	7
	41+	17	34	4	20	13	43
**Weight (kg)**							
	50-60	14	28	6	30	8	27
	61-75	24	48	9	45	15	50
	≥76	12	24	5	25	7	23
**Height (cm)**							
	150-160	2	4	0	0	2	7
	161-170	18	36	9	45	9	30
	171-180	16	32	5	25	11	37
	≥181	14	28	6	30	8	27
**Years been running** ^a^							
	≤6	21	42	9	45	12	40
	≥8	29	58	11	55	18	60
**Kilometers run per week** ^a^							
	15-30	23	46	10	50	13	43
	31-40	14	28	5	25	9	30
	≥ 41	13	26	5	25	8	27

^a^As the participants were required to describe the average distance run per week and years of running experience within certain categories, for example, 15-20 km/week, 10+ years, no means or SD could be calculated for these variables.

### Participants in Group 2

There were 30 participants that fulfilled group 2 criteria by completing the survey on the Internet then again by phone interview within 14 days after Internet completion. Participants were between 18 and 67 years of age, with an almost equal distribution of males (53%, n=16) and females (47%, n=14). There were 40% (12/30) of participants that had been running for 6 years or less and the remaining 60% (18/30) 10 years or more. Their running habits were similar to test group 1 with 43% (13/30) predominantly running 15-30 km/week, 30% (9/30) 30-40 km/week, and 27% (8/30) 40 km or more per week (Table 2).

### Test Group 1

Reliability was almost perfect for the type of terrain run on (kappa=.863), injuries of the lower limbs reported in the last 2 years (kappa>.99), and the number of these lower limb injuries diagnosed in the same time frame (kappa>.99). Reliability was also almost perfect for the injury being diagnosed by a doctor (kappa=.820), the injury being a bone stress injury (kappa>.99), reporting any hip/knee/ankle surgery (kappa=.857), and the use of orthotics while running (kappa>.99). Reliability was substantial for the number of years running on a regular basis (kappa=.791), average kilometer run per week (kappa=.684), other sports or intentional exercise participated in regularly in the last 2 years (kappa=.773), and reporting the injury as an Achilles tendon injury (kappa=.625). Reliability was moderate for being diagnosed by a physiotherapist (kappa=.467; Table 3).

**Table 3:** Test group 1 (Internet-Internet), test-retest reliability for running habits, injuries, and general health.

Test-retest reliability		n	Number of response options	Kappa (A-symp SE)
**Running habits and injuries**				
	How many years have you been running on a regular basis? Regular is defined as at least weekly.	20	11	.791 (.100)
	On average, how many kilometer per week would you run?	20	7	.684 (.119)
	What type of terrain is the majority of your running performed on?	20	7	.863 (.088)
	In the last 2 years, have you participated in any other sports or intentional exercise on a regular basis (eg, weekly during at least one season)?	20	2	.773 (.216)
	In the last 2 years, have you had any injuries of the lower limbs, which have forced you to discontinue running for a period of 2 weeks or more?	20	2	>.99
	If yes, how many lower limb injuries have you been diagnosed with in the last 2 years?	12^a^	4	>.99
	Was the injury diagnosed by a professional doctor?	11^b^	2	.820 (.169)
	Was the injury diagnosed by a professional physiotherapist?	8^c^	2	.467 (.323)
	Was the injury an Achilles tendon injury?	12^a^	2	.625 (.333)
	Was the injury a bone stress injury below the knee?	12^a^	2	>.99
	Have you ever had hip, knee, or ankle surgery?	20	2	.857 (.138)
	While running do you wear orthotics?	20	2	>.99
**General health**				
	Have you ever smoked cigarettes?	19^d^	2	>.99
	To your knowledge, have you ever been treated using quinolone antibiotics (eg, ciprofloxacin, norfloxacin)?	20	3	.468 (.174)
	To your knowledge, have you ever been treated using corticosteroid medication?	20	3	.492 (.165)
	To your knowledge, have you ever been treated using calcium tablets as prescribed by a medical doctor or taken it without a prescription?	20	3	.886 (.110)
	To your knowledge, have you ever been treated using vitamin D supplementation as prescribed by a medical doctor or taken it without prescription?	20	3	>.99
	Do you follow a gluten-free diet?	20	2	>.99
	Do you have any food allergies or avoidances?	20	2	>.99

^a^n=20; valid=12; excluded=8

^b^n=20; valid=11; excluded=9

^c^n=20; valid=8; excluded=12

^d^n=20; valid=19; excluded=1

### Reliability for General Health Questions

Reliability was almost perfect for the majority of all general health questions reported including having ever smoked cigarettes (kappa>.99), any knowledge of being treated with calcium tablets (kappa=.886), any knowledge of being treated with vitamin D supplementation (kappa>.99), reporting on whether participants followed a gluten-free diet (kappa>.99), or stating any food allergies or avoidances (kappa>.99). Reliability for any knowledge of being treated with corticosteroid medication (kappa=.492) or quinolone (kappa=.468) was moderate (Table 3).

### Test Group 2

The reliability of running habit and running injury key variables in test group 1 was more variable than that of test group 1 (Table 4). Reliability was almost perfect for injuries of the lower limbs in the last 2 years, which forced discontinuation of running for a period of 2 weeks or more (kappa=.930), reporting the injury as an Achilles tendon injury (kappa=.814), reporting any hip/knee/ankle surgery (kappa>.99), and the use of orthotics while running (kappa>.99). Reliability was substantial for the number of years running on a regular basis (kappa =.639), the number of lower limb injuries diagnosed within the last 2 years (kappa=.697), injury diagnosed by a doctor (kappa=.727), and injury diagnosed by a physiotherapist (kappa=.615). Reliability was moderate for kilometer run per week (kappa=.540), the type of terrain run on (kappa=.469), other sports or intentional exercise participated in regularly in the last 2 years (kappa=.473), and reporting the injury as bone stress injury (kappa=.571; Table 4).

**Table 4:** Test group 2 (Internet-phone), test-retest reliability for running habits, injuries, and general health.

Test-retest reliability		n	Number of response options	Kappa (A-symp SE)
**Running habits and injuries**				
	How many years have you been running on a regular basis? Regular is defined as at least weekly.	30	11	.639 (.102)
	On average, how many kilometer per week would you run?	30	7	.540 (.108)
	What type of terrain is the majority of your running performed on?	30	7	.469 (.129)
	In the last 2 years, have you participated in any other sports or intentional exercise on a regular basis (eg, weekly during at least one season)?	29^a^	2	.473 (.306)
	In the last 2 years, have you had any injuries of the lower limbs, which have forced you to discontinue running for a period of 2 weeks or more?	30	2	.930 (.069)
	If yes, how many lower limb injuries have you been diagnosed with in the last 2 years?	10^b^	4	.697 (.187)
	Was the injury diagnosed by a professional doctor?	9^c^	2	.727 (.247)
	Was the injury diagnosed by a professional physiotherapist?	10^b^	2	.615 (.337)
	Was the injury an Achilles tendon injury?	11^d^	2	.814 (.175)
	Was the injury a bone stress injury below the knee?	6^e^	2	.571 (.353)
	Have you ever had hip, knee, or ankle surgery?	30	2	>.99
	While running do you wear orthotics?	30	2	>.99
**General health**				
	Have you ever smoked cigarettes?	30	2	.839 (.157)
	To your knowledge, have you ever been treated using quinolone antibiotics (eg, ciprofloxacin, norfloxacin)?	27^f^	3	.150 (.132)
	To your knowledge, have you ever been treated using corticosteroid medication?	30	3	.583 (.124)
	To your knowledge, have you ever been treated using calcium tablets as prescribed by a medical doctor or taken it without a prescription?	30	3	.720 (.184)
	To your knowledge, have you ever been treated using vitamin D supplementation as prescribed by a medical doctor or taken it without prescription?	30	3	.672 (.170)
	Do you follow a gluten-free diet?	30	2	.783 (.209)
	Do you have any food allergies or avoidances?	29^a^	2	.922 (.077)

^a^n=30; valid=29; excluded=1

^b^n=30; valid=10; excluded=20

^c^n=30; valid=9; excluded=21

^d^n=30; valid=11; excluded=19

^e^n=30; valid=6; excluded=24

^f^n=30; valid=27; excluded=3

### Reliability of Test Group 1 Compared to Test Group 2

Overall, reliability was lower for general health questions reported in test group 2 in comparison to test group 1 (Table 4). Reliability was almost perfect for having ever smoked cigarettes (kappa=.839), and stating any food allergies or avoidances (kappa=.922). Reliability for any knowledge of being treated with calcium tablets (kappa=.720), any knowledge of being treated with vitamin D supplementation (kappa=.672), and reporting on whether participants followed a gluten-free diet (kappa=.783) was substantial. Reliability was moderate for any knowledge of being treated with corticosteroid medication, however, any knowledge of being treated with quinolone had slight reliability (kappa=.150; Table 4).

## Discussion

### Principal Findings

The wider study, for which this survey will provide epidemiology information and a participant cohort to recruit from, will be the first, to our knowledge, to examine the genetic predisposition of tendon and bone injury in adult recreational runners. Recruitment for the larger study will involve the use of this survey, tested here initially for test-retest reliability. The results indicate that the self-reported questionnaire, delivered using the Internet commercial software, provided stable and reliable answers for many of the most important measures required for recruiting to the larger study on tendon and bone injury.

The Internet delivery of a retrospective injury study is a convenient method for remote collection of data from participants and has been used successfully in the analysis of sports-induced injuries in surfing [16] and strongman athletes [18]. As mentioned, the survey developed here combined new items of assessment with adapted versions of the SCOFF questionnaire for examining eating disorders [17]. In most aspects, its reliability was comparable to that of other similar survey delivery methods used for examining health and injury-related factors, including the Military Pre-training Questionnaire, which assesses risk factors for injury among military trainees across 5 domains (physical activity, injury history, diet, alcohol, and smoking) [19]. Test-retest reliability, for the study reported here, was shown to be acceptable for all variables for all participants, supporting the stability of the questionnaire. The only variable questionable for reliability was “treated using quinolone” (kappa=.150) for test group 2. On first completion of the Internet survey, there were 10 participants (n=30; 27 valid; 3 excluded) that were “unsure” if they had been treated in the past with quinolone. In the phone interview, these same 10 participants reported that they had not been treated previously with quinolone, and therefore this shift in reporting is responsible for the low reliability.

Poor reliability of the question addressing treatment with quinolone may suggest poor understanding of or unfamiliarity with “quinolone” upon first exposure to the question. It is known that test-retest reliability can be influenced by many factors. Interpretation of a question, such as familiarity of content or ambiguity, as well as memory can cause random answers [20]. Questions which involve unfamiliar knowledge or are ambiguous, such as asking about treatment with various prescribed medications in this survey, may lead to considerable variability in test-retest responses [21]. The nature of the question can also shift test-retest reliability scores. As we report here, it is not unusual that constant behaviors, such as smoking, result in higher reliability than more variable behavior such as obtaining an injury [11,12], or being diagnosed or treated with different medications over time [22]. Responses to questions inquiring about variable behaviors, including those addressing all aspects of running habits, would probably be more reliable if a training diary was referred to when obtaining data rather than relying on retrospective recall. However, the data presented in our study indicate moderate to almost perfect reliability for questions asking about variable behaviors without requiring participants to refer to such a tool.

Respondent fatigue is a well-known phenomenon affecting survey participants [23]. We developed this survey to minimize such fatigue by making the questions as easy to understand and answer as possible, in addition to being mindful of the time required to complete the survey. Questions regarding a gluten-free diet and known food allergies were among some of the last questions addressed in survey. We show high reliability for these variables, which supports our belief of minimal user fatigue across the questionnaire in both test groups (Tables 3 and 4). Increasing the number of possible responses did not markedly affect the reliability of the questions. Responses for questions containing only 2 variables (yes/no answers) had perfect or high reliability, while questions with 7 or 11 possible responses had moderate to high variability. This is consistent with previous research demonstrating that increasing the number of response categories only negligibly affects reliability [24,25].

Results when addressing the 2 different test groups should be considered in the context of several limitations. Our study contained a different number of participants in each group with a variable distribution of age. This limitation resulted from the sequential manner of recruitment of the two groups, inviting participation from within our networks for test group 1, then soft launching to recruit a larger group from the running club for test group 2.

There was a slightly higher test-retest reliability for test group 1, in which the survey was repeated using the same Internet platform. It is possible that this was due to learning effects, or that the retest was administered in exactly the same format.

The slightly lower test-retest reliability for group 2, in which the survey was administered by the Internet platform and then followed up by a telephone call, may be related to the wording of the question over the telephone by the interviewer compared to the participants reading the computer screen. During the telephone assessment, it is also possible that participants felt more uncomfortable answering sensitive questions or got confused in regards to what constitutes a response for each question asked. Our findings indicate that using 2 test-retest protocols resulted in participants providing the same information with high reliability for the survey administered in this study. Maintaining consistency of the protocol, however, can improve test-retest reliability.

### Conclusions

We successfully developed an Internet survey to meet the recruitment needs of the larger genetic injury study. We tested its reliability using 2 different test-retest methods and used a participant sample targeted to our needs (adult recreational runners) in aid of focusing our findings to support our larger study. We had equal gender participation and a broad range of running habits and participants with reportable Achilles and bone stress injuries. Despite the aforementioned limitations, the results of this reliability study demonstrate that the Internet survey developed does provide a relatively quick, easy to complete, and cost-effective method to collect epidemiological data from recreational runners and evaluate these participants for inclusion into a genetic study.

## Appendix

Multimedia Appendix 1 Survey: The genetics of exercise-induced injuries in tendon and bone.
