# Supplementary material for: Exercise-Induced Tendon and Bone Injury in Recreational Runners: A Test-Retest Reliability Study
Source: JMIR Res Protoc. 2015 Oct 7;4(4):e117. doi: 10.2196/resprot.4585 (PMC6858051; doi:10.2196/resprot.4585)
Supplement: Multimedia Appendix 1 [file jmir_v4i4e117_app1.pdf]

The genetics of exercise-induced injuries in tendon and bone

Welcome!

- **You are invited to take part in this research project if you are:**
  - **A recreational runner**
  - **Aged 18 and over**
  - **Run over 15 km per week**
- **Please read the Participant Information Form carefully as this will tell you about the research project and explain what is involved. This will help you decide if you want to continue and take part.**
- **Ask questions about anything that you don't understand or want to know more about. Before deciding whether or not to take part, you might want to talk about it with a relative, friend or healthcare worker.**

<u>**Participation**</u>

- **Participation in this study is entirely voluntary.**
- **You're not obliged to participate and if you do, you can withdraw at any time without penalty or prejudice.**
- **To participate, we would like you to complete this online questionnaire, providing details of your medical history, injury history and running habits.**
- **This survey should take no more than 30 minutes to complete.**
- **You are able to exit the survey and complete at a later date using the link at the top of the page.**
- **Your participation, personal details and results will be strictly confidential and only the principal researchers above will have access to this information**

  By ticking the 'I ACCEPT' option below you are telling us that you:

- **understand what you have read;**
- **consent to take part in the research project;**
- **consent to participate in the research processes that are described;**
- **consent to the use of your personal and health information as described;**
- **understand that you are free to not answer specific items or questions in interviews or questionnaires;**
- **understand that any data or answers to questions will remain confidential with regard to your identity;**
- **certify to the best of your knowledge and belief, you have no physical or mental illness or weakness that would increase the risk of participating in this project;**
- **are participating in this project of your own free will and have not been coerced in any way to participate.**

Do you accept to participate in this research study?*

( ) **I ACCEPT** the conditions above

( ) **I DO NOT ACCEPT** the conditions above

Further information about the project is available in the Participant Information Form

[ ] I wish to see more information about the project:

Your Personal Details

1) Please enter your contact information here:

First Name*: ____

Last Name*: ____

Street Address: ____

City: ____

State

( ) ACT

( ) NSW

( ) NT

( ) QLD

( ) SA

( ) TAS

( ) VIC

( ) WA

Postcode: ____

Email Address*: ____

Phone Number: ____

2) Please enter your personal details here

What year were you born?*: ____

What month were you born?

( ) January

( ) February

( ) March

( ) April

( ) May

( ) June

( ) July

( ) August

( ) September

( ) October

( ) November

( ) December

What date of the month were you born?

( ) 1

( ) 2

( ) 3

( ) 4

( ) 5

( ) 6

( ) 7

( ) 8

( ) 9

( ) 10

( ) 11

( ) 12

( ) 13

( ) 14

( ) 15

( ) 16

( ) 17

( ) 18

( ) 19

( ) 20

( ) 21

( ) 22

( ) 23

( ) 24

( ) 25

( ) 26

( ) 27

( ) 28

( ) 29

( ) 30

( ) 31

Sex*
( ) Male

( ) Female

Weight (kgs)*: ____

Height (cm)*: ____

I give permission, if I am eligible, to be contacted in the future for related research*

( ) Yes

( ) No

I give permission, if I am eligible, to be contacted in the future to provide a saliva sample for genetic related analysis*

( ) Yes

( ) No

Your Ethnic Background

3) What is your country of birth?

( ) Australia

( ) Other: ____

4) What is your country of citizenship?

( ) Australia

( ) Other: ____

5) What is the ethnic background of your biological grandparents?

Maternal Grandmother, Maternal Grandfather, Paternal Grandmother, Paternal Grandmother
Answer options:
( ) Caucasian European
( ) Mediterranean
( ) Asian
( ) African
( ) Polynesian
( ) Indigenous Australian or TSI
( ) Unknown
( ) Other

Your Running Habits

6) Which is your dominate leg* (used for kicking a ball)? *If you are unsure please choose according to whether you are left or right handed

( ) Left

( ) Right

( ) Ambidextrous

7) How many years have you been running on a regular* basis? *Regular is defined as at least weekly.

	**< 1**	**1**	**2**	**3**	**4**	**5**	**6**	**7**	**8**	**9**	**10 +**
Years	( )	( )	( )	( )	( )	( )	( )	( )	( )	( )	( )

8) On average, how many km per week would you run?

	**<15**	**15-20**	**20-30**	**30-40**	**40-50**	**50-60**	**60 +**
Km	( )	( )	( )	( )	( )	( )	( )

9) Do you run every day?

( ) Yes

( ) No

10) For an average week, please indicate how many running sessions you participate in.

	**1**	**2-3**	**4-5**	**6+**
sessions/week	( )	( )	( )	( )

11) What type of terrain is the majority of your running performed on?
( ) Bitumen

( ) Cement

( ) Hard dirt or gravel

( ) Sand

( ) Grass

( ) Synthetic

( ) Treadmill

12) What is your current race pace in min/km?

	**> 7**	**6-7**	**5-6**	**4-5**	**< 4**
min/km	( )	( )	( )	( )	( )

13) Do you spend time stretching in association with your running session?
( ) Yes

( ) No

14) If Yes, when do you stretch?
( ) Before running

( ) After running

( ) Both before and after running

15) While running do you wear orthotics?
( ) Yes

( ) No

16) If Yes, which foot?
( ) Left foot

( ) Right foot

( ) Both feet

17) If Yes, are they custom made?
( ) Yes

( ) No

18) What proportion of your running is: (please ensure that total of all entries equals 100%)
*minimalist means with no support or cushioning, e.g. aqua shoes, vibram five fingers (does not include Nike free, etc.)

________with standard running shoes?

________with minimalist running shoes?

________barefoot?

19) In the last two years have you participated in any other sports or intentional exercise on a regular basis (for example weekly during at least one season)?
( ) Yes

( ) No

20) If yes, what sports? (please list all)
____

Running Related Injuries

21) In the **last 2 years** have you had any injuries of the lower limbs, which have forced you to discontinue running for a period of 2 weeks or more?*

( ) Yes

( ) No

22) If Yes, How many lower limb injuries have you been diagnosed with in the past 2 years?

( ) 1

( ) 2

( ) 3

( ) 4 +

Injury 1 - Your Most Recent Injury

Please answer the following questions in relation to **your most recent lower limb injury.**

23) You indicated that you have been diagnosed with a lower limb injury within the past 2 years How did this injury occur?

( ) while running

( ) while walking

( ) due to a fall

( ) during participation in another sport

( ) other

24) Was this injury diagnosed by a professional:

	**Yes**	**No**
Doctor?	( )	( )
Physical Therapist?	( )	( )

25) Was this injury diagnosed by imaging (x-ray/ultrasound/bone scan/CT scan/MRI)?

( ) Yes

( ) No

26) If imaging, what type of diagnostic imaging?

( ) x-ray

( ) ultrasound

( ) bone scan

( ) CT scan

( ) MRI

27) Do you have a copy of the report of the imaging findings?
( ) Yes

( ) No

28) Was the injury an Achilles tendon injury?
( ) Yes

( ) No

29) If Yes, which leg?
( ) Right leg

( ) Left leg

( ) Both legs

30) Was the injury a bone stress injury below the knee?
( ) Yes

( ) No

31) If yes, which leg?
( ) Right leg

( ) Left leg

( ) Both legs

32) Was your injury a different injury (other than an Achilles tendon or bone stress injury)?
( ) Yes

( ) No

33) What type of injury was it?
____

34) Please provide details of the signs and symptoms of the injury (tick all that apply):
[ ] bleeding

[ ] laceration

[ ] swelling

[ ] bruising

[ ] tenderness to touch

[ ] pain at rest

[ ] pain on movement

[ ] instability of joint

[ ] weakness

[ ] numbness

[ ] loss of sensation

[ ] other

35) When is/was your pain at its worst?
( ) pain during warm up

( ) pain during exercise

( ) pain after exercise

( ) pain at night

( ) unable to exercise due to pain

36) As a result of this injury, how many weeks did you discontinue running?

	**1**	**2**	**3**	**4**	**5**	**6**	**6 +**
weeks	( )	( )	( )	( )	( )	( )	( )

37) How and when did your symptoms start?
( ) sudden onset after injury

( ) gradual onset of pain

( ) pain during running

( ) other

38) What type of treatment did you have in association with this injury?
( ) Rest only

( ) Other treatment

39) What type of treatment did you have in association with this injury?
[ ] medication

[ ] physical therapy

[ ] surgery

[ ] bracing/taping

40) If medication please provide details

____

41) Had you made any changes to your regular training program just prior to the onset of injury?
(for example, increase in training load, change in footwear, change in terrain)
( ) Yes

( ) No

42) If yes, please provide details:

____

43) Had you begun or changed your participation in any new exercise other than running prior to the injury?
(for example basketball, touch football, tennis, another sport etc.)
( ) Yes

( ) No

44) If yes, please provide details:

____

45) Please upload any associated medical reports in relation to this injury.

You may upload a scanned report/image or a smartphone picture of the report/image (*accepted file types include png, jpg, doc, xls, docx, xlsx, pdf, txt maximum file size 1 MB*).

________1

46) If you do not have the medical reports, do you give us permission to contact your health care provider to obtain reports relevant to this injury?

( ) Yes

( ) No

( ) N/A

47) Please provide name and contact details of the medical provider who holds these records.

We will send you a permission slip to sign and a stamped envelope addressed to this medical provider.
This is necessary for the release of your records to us for this survey.

Name of medical provider:: ____

Address of medical provider:: ____

Phone contact for medical provider:: ____

Injury 2 - Your Second Most Recent Injury

Please answer the following questions in relation to **your second most recent lower limb injury.**
Repeated injury questions, see questions 23 - 47

Re-occurring Injury

73) You have indicated that you have had more than one lower limb injury.

Was the diagnosis of the 2nd injury the same as the first injury?

( ) Yes

( ) No

74) If yes, was it the same foot/leg?

( ) Yes

( ) No

75) How many days, weeks, months passed between injuries?

Days: ____

Weeks: ____

Months: ____

76) Were the symptoms of the second injury the same, better or worse than the first injury?

( ) Same

( ) Better

( ) Worse

77) Was the treatment the same for both injuries?
( ) Yes

( ) No

78) Was the recovery longer, shorter or similar in duration?
( ) Longer

( ) Shorter

( ) Similar

79) Do you have a family history of exercise-related or other lower extremity injury?
( ) Yes

( ) No

80) If yes, was it:

	**Yes**	**No**
bone stress?	( )	( )
Achilles tendinopathy?	( )	( )
another injury?	( )	( )

81) What family member did it occur in?
- check as many as relevant

[ ] Maternal Grandmother

[ ] Maternal Grandfather

[ ] Paternal Grandmother

[ ] Paternal Grandfather

[ ] Mother

[ ] Father

[ ] Brother

[ ] Sister

[ ] Maternal Aunt

[ ] Paternal Aunt

[ ] Maternal Uncle

[ ] Paternal Uncle

[ ] Maternal 1st Cousin

[ ] Paternal 1st Cousin

Injury 3 - Your Third Most Recent Injury
Please answer the following questions in relation to **<u>your third most recent lower limb injury.</u>**

Repeated injury questions, see questions 23 - 47

Injury 4 - Your Fourth Most Recent Injury
Please answer the following questions in relation to **your fourth most recent lower limb injury.**
Repeated injury questions, see questions 23 - 47

General Health Questions
132) Have you ever smoked cigarettes?
( ) Yes

( ) No

133) If Yes, at what age did you start smoking?

____

134) Do you currently smoke?
( ) Yes

( ) No

135) If Yes, how many cigarettes per day on average?

____

136) If No, at what age did you quit?

____

137) When you were smoking, on average how many cigarettes per day would you smoke over the years until you quit?

____

138) Do you ever consume alcoholic drinks?
( ) Yes

( ) No

139) If yes, approximately how many standard drinks would you consume per week? Examples of standard drinks include 100ml of wine, a bottle of mid-strength beer, a 285ml glass(midi) of full strength beer or a 30ml nip of spirits.

	**1-3**	**4-6**	**7-10**	**10 +**
drinks	( )	( )	( )	( )

General Health Questions
140) Have you ever been diagnosed with any of the following conditions/disorders?

	**Yes**	**No**
any type of cancer	( )	( )
chronic renal failure	( )	( )
rheumatoid arthritis	( )	( )

osteoarthritis	( )	( )
osteoporosis	( )	( )
diabetes	( )	( )
cystic fibrosis	( )	( )
cerebral palsy	( )	( )
cardiac conditions	( )	( )
high blood pressure	( )	( )
anaemia	( )	( )
skin diseases	( )	( )
thyroid disease	( )	( )
gastrointestinal disease	( )	( )
depression	( )	( )
insomnia	( )	( )
respiratory conditions	( )	( )
neurological conditions	( )	( )
other	( )	( )

141) Have you ever had hip, knee or ankle surgery?
( ) Yes

( ) No

142) If yes,

	**Yes**	**No**
Hip	______________________	______________________
Knee	______________________	______________________
Ankle	______________________	______________________

143) Have you ever had a fracture of any bone?
( ) Yes

( ) No

144) If yes:
Where was the fracture?: ____

When did it occur?: ____

What treatment did you receive?: ____

145) Do you have a family history of osteoporosis?
( ) Yes

( ) No

( ) Unsure

146) To your knowledge, have you ever been treated using quinolone antibiotics (for example ciprofloxacin, norfloxacin)?
( ) Yes

( ) No

( ) Unsure

147) If yes, were you treated using these antibiotics within the 6 months prior to your injury?
( ) Yes

( ) No

( ) Unsure

148) To your knowledge, have you ever been treated using anti-seizure or epilepsy medications (for example clonazepam, gabapentin, lamotrigine, sodium valproate)?
( ) Yes

( ) No

( ) Unsure

149) To your knowledge, have you ever been treated using corticosteriod medication (for example cortisone injection, prednisone tablets, prednisolone tablets, flixotide inhaler, pulmicort inhaler, QVAR inhaler, seretide accuhaler, symbicort turbuhaler, steriod cream)?
( ) Yes

( ) No

( ) Unsure

150) If yes, how was this drug administered?
[ ] Injection

[ ] Topical Cream

[ ] Tablet

[ ] Inhaler

151) Which type of inhaler?
____

General Health Questions
152) To your knowledge, have you ever been treated using calcium tablets as prescribed by a medical doctor or taken it without a prescription?
( ) Yes

( ) No

( ) Unsure

153) Do you take calcium tablets on a regular basis?
( ) Yes

( ) No

154) If yes, please provide details of dose and brand:
Dose: ____

Brand: ____

155) To your knowledge, have you ever been treated using vitamin D supplementation as prescribed by a medical doctor or taken it without prescription?

( ) Yes

( ) No

( ) Unsure

156) Do you take vitamin D supplementation on a regular basis?

( ) Yes

( ) No

157) If yes, please provide details of dose and brand:

Dose: ____

Brand: ____

158) To your knowledge, have you ever

	Yes	No	Unsure
been treated using bisphosphonates (for example actonel, Didrocal, alendronate sodium, zoledronic acid)?	( )	( )	( )
undergone chemotherapy?	( )	( )	( )
undergone a bone marrow or organ transplant?	( )	( )	( )

159) Are you regularly taking, or have you ever taken, any other regular medication as prescribed by a medical doctor?

( ) Yes

( ) No

160) If yes, please list medications

____

161) Are you regularly taking, or in the past have you regularly taken any other over-the-counter medications, dietary supplements or sports supplements?

( ) Yes

( ) No

162) If yes, please list any other medications/supplements

[ ] B-alanine

[ ] B-Vitamins

[ ] Creatine

[ ] Fish Oil

[ ] Glucosamine

[ ] Iron

[ ] Probiotics

[ ] Protein Powder

[ ] Vitamin C

[ ] Zinc

[ ] Other: ____

Dietary Habits

163) Would you say that food dominates your life?

( ) Yes

( ) No

164) Do you:

	**Yes**	**No**
Currently suffer with or have you ever suffered in the past with an eating disorder?	( )	( )
Make yourself sick because you feel uncomfortably full?	( )	( )
Worry you have lost control over how much you eat?	( )	( )
Believe yourself to be fat when others say you are too thin?	( )	( )

165) Have you recently lost more than 6kgs in a 3 month period?

( ) Yes

( ) No

166) Have you undergone any significant (greater than 5 kg) weight gain or loss in the past 2 years?

( ) Yes

( ) No

167) If Yes, please indicate the amount of weight gained/lost

weight gain (kgs): ____

weight lost (kgs): ____

reason (if any known): ____

168) Do you follow any of the diets below:

	**Yes**	**No**
Vegetarian?	( )	( )
Lacto-ovo vegetarian?	( )	( )
Pesco-vegetarian?	( )	( )
Vegan?	( )	( )
Paleolithic/Paleo?	( )	( )
Low carb/High Fat?	( )	( )

169) Do you follow a gluten free diet?

( ) Yes

( ) No

170) If yes, please provide the reason why you follow this diet

( ) Coeliac disease

( ) Medically diagnosed gluten intolerance

( ) Undiagnosed gluten intolerance

( ) Irritable bowel syndrome

( ) Other

171) Do you have any food allergies or avoidances?

( ) Yes

( ) No

172) If yes,

	**Food type**	**Reason for avoidance**
1	____	____
2	____	____
3	____	____
4	____	____

Female Health Questions

173) Are you currently taking or have you ever taken the contraceptive pill?

( ) Yes

( ) No

174) If yes, how long have you or did you take the contraceptive pill?

	**< 1 year**	**1-2 years**	**3-5 years**	**5 + years**
Length of time	( )	( )	( )	( )

175) What was the name of the contraceptive pill you were taking?

____

176) Do you have any children?
( ) Yes

( ) No

177) Please indicate the year of birth for each child
Child 1: ____

Child 2: ____

Child 3: ____

Child 4: ____

Child 5: ____

178) What is the regular length of your menstrual cycle?

	**I don't have a period**	**Irregular**	**< 26 days**	**27 - 31 days**	**> 31 days**
Cycle length	( )	( )	( )	( )	( )

179) Please provide a reason why you don't have a period:
____

180) Have you gone through menopause?
( ) Yes

( ) No

181) If yes, at what age did you go through menopause?
____

182) Was the menopause:
( ) Natural

( ) Surgical

183) Have you experienced amenorrhea (absence of menstrual periods) or oligomenorrhea (infrequent or irregular menstruation) or menorrhagia (excessive menstruation)?
( ) Yes

( ) No

184) What age did you commence your menstrual period?

	**Early than 12 years old**	**12 years old**	**13 years old**	**14 years old**	**15 years old**	**Later than 15 years old**
Age	( )	( )	( )	( )	( )	( )

Complete
Thank You for Participating!

If you have indicated and are eligible, you may be contacted in the future to provide a saliva

sample for analysis.
